# Diameter-selective extraction of single-walled carbon nanotubes by interlocking with Cu-tethered square nanobrackets

**DOI:** 10.3762/bjoc.20.113

**Published:** 2024-06-05

**Authors:** Guoqing Cheng, Naoki Komatsu

**Affiliations:** 1 Graduate School of Human and Environmental Studies, Kyoto University, Sakyo-ku, Kyoto 606-8501, Japan

**Keywords:** complexation, diameter, extraction, interlock, nanobracket, single-walled carbon nanotubes, separation

## Abstract

We have been working with carbon nanotube separation through host–guest chemistry. Herein, a new macrocyclic host molecule, Cu-tethered square nanobrackets, is designed, synthesized and applied to single-walled carbon nanotubes (SWNTs) for their diameter-based separation. The complexation between copper ions and dipyrrin moieties of the nanobracket gives Cu-tethered square nanobrackets, which is confirmed by absorption, Raman and MALDI-TOF mass spectra. Upon extraction of SWNTs with the nanobracket and copper(II), in situ-formed square Cu-nanobrackets are found to interlock SWNTs to disperse them in 2-propanol. The interlocking is confirmed by Raman spectroscopy after thorough washing of the extracted SWNTs. Pristine SWNTs were recovered through demetalation of the interlocked ones along with the nanobracket. Raman and absorption spectroscopies of the extracted SWNTs reveals the diameter enrichment of only several kinds of SWNTs in the diameter range from 0.94 to 1.10 nm among ≈20 kinds of SWNTs from 0.76 to 1.20 nm in their diameter range. The diameter selectivity is supported by the theoretical calculations with the GFN2-xTB method, indicating that the most preferred SWNT diameter for the square Cu-nanobrackets is 1.04 nm.

## Introduction

Single-walled carbon nanotubes (SWNTs) have been attracting considerable interest due to their unique physical and chemical properties. For their further applications, structural control of SWNTs is of great importance, since the electronic and optical properties significantly depend on their structures [1]. To obtain specific structures, remarkable progress has been made in separation of SWNTs by various methods such as gel column chromatography [2–3], aqueous two-phase extraction (ATPE) [4–5], and polymer wrapping [6–7]. As compared with these methods, our separation by host–guest chemistry has advantages in terms of the rational design of the host molecules to target specific structures and the thorough removal of the dispersants, host molecules in our case, to recover pristine carbon nanotubes (CNTs) after separation. Moreover, the association constants of the complexation between host molecules and SWNTs are strongly related to the molecular structures, making it possible to tune the binding affinity [8].

**Scheme 1:**
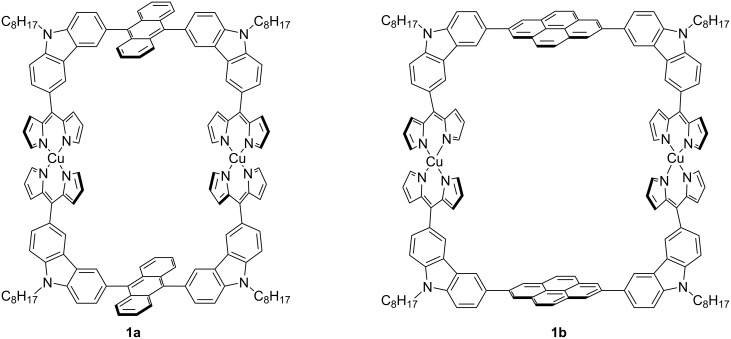
Chemical structures of Cu-tethered tetragonal nanobrackets **1a** and **1b**.

The host molecules we employed so far for CNT separation through molecular recognition have been developed as “nanotweezers” [9], “nanocalipers” [10] and, recently, a rigid metal-tethered macrocyclic host molecule “M-nanobrackets” (M = Cu, Co and Pd) [11]. While the concept of the mechanically interlocked carbon nanotubes (MINTs) has been established by Pérez and López [12–13], Cu-tethered tetragonal nanobrackets **1a** not only interlocked SWNTs, but also selectively extracted a narrow diameter range (0.90–0.92 nm) and released them through demetalation (Scheme 1).

Since one of the advantages of our method is rational design of host molecules as mentioned above [14–15], the cavity of the Cu-nanobrackets **1b** is enlarged by ≈0.2 nm in its size and changed to square in its shape by changing anthracene in **1a** to pyrene in **1b**. To interpret the results of the diameter selectivity, the theoretical calculation using the semiempirical tight binding quantum chemical method GFN2-xTB was employed instead of the molecular mechanics used in our previous works, because it can calculate larger system (>500 atoms) containing transition metals more precisely [16–17].

## Result and Discussion

### Synthesis of Cu-tethered square nanobrackets **1b**

**Scheme 2:**
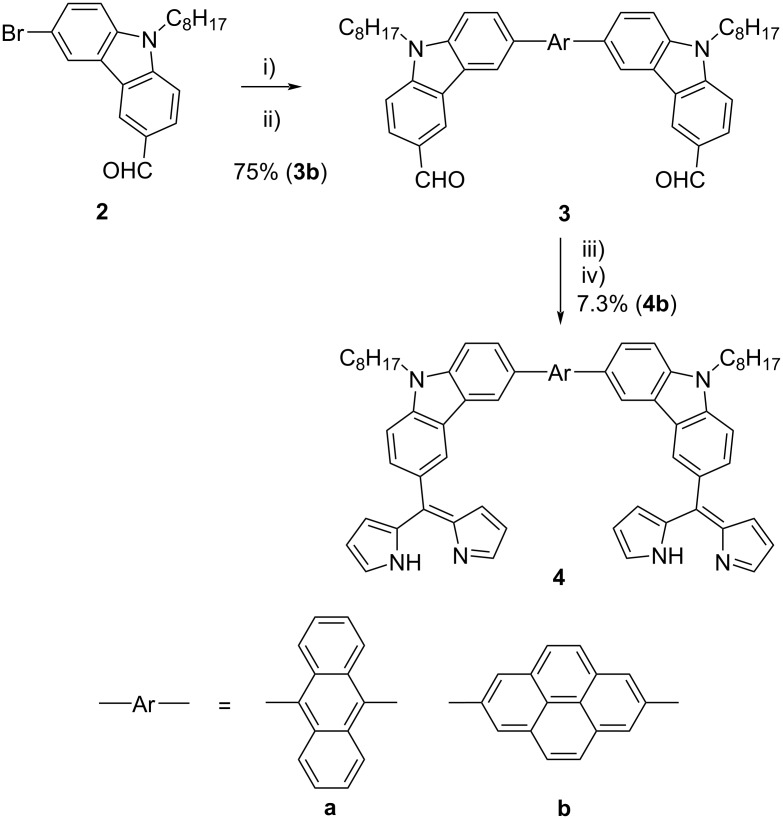
Synthesis of nanobracket **4**. Reaction conditions: i) XPhos Pd G2, XPhos, B_2_(OH)_4_, KOAc, EtOH, 80 °C, 2 h; ii) Br-Ar-Br, K_2_CO_3_, tetrahydrofuran (THF)/toluene, 80 °C, 16 h; iii) trifluoroacetic acid (TFA), pyrrole, rt, 1.5 h; iv) *p*-chloranil, dichloromethane, rt, 5 h.

A pyrene-based nanobracket **4b** was synthesized via a one-pot Suzuki coupling followed by the formation of dipyrrin from the dialdehyde **3** (Scheme 2) [11]. The products were fully characterized by ^1^H and ^13^C NMR, and MALDI-TOF mass spectroscopy (see Supporting Information File 1). The total yield of **4b** (5.5%) based on 2,7-dibromopyrene is much lower than that of **4a** (44%) [11], because a larger amount of the product **4b** was lost in the chromatographic purification due to much lower solubility of **4b** compared to **4a**.

**Figure 1:**
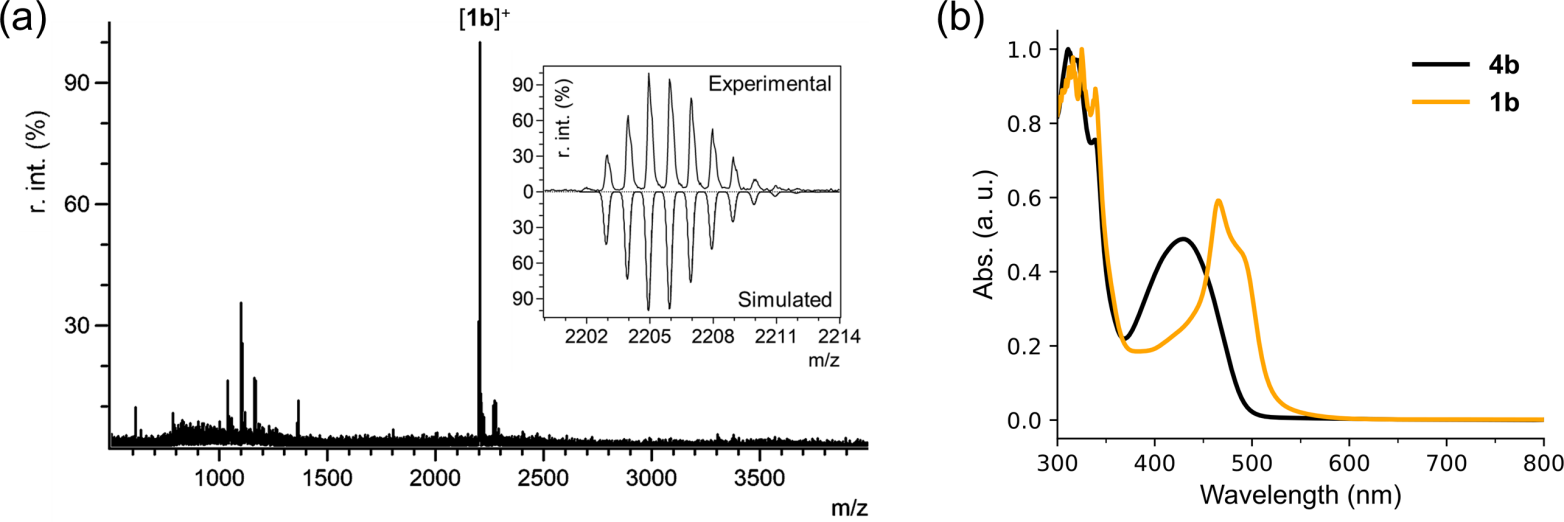
(a) MALDI-TOF mass spectrum of Cu-nanobrackets **1b**, where the inset shows the isotope peaks of [**1b**]^+^ comparing with the simulated ones; (b) absorption spectra of the nanobracket **4b** and Cu-nanobrackets **1b**.

**Figure 2:**
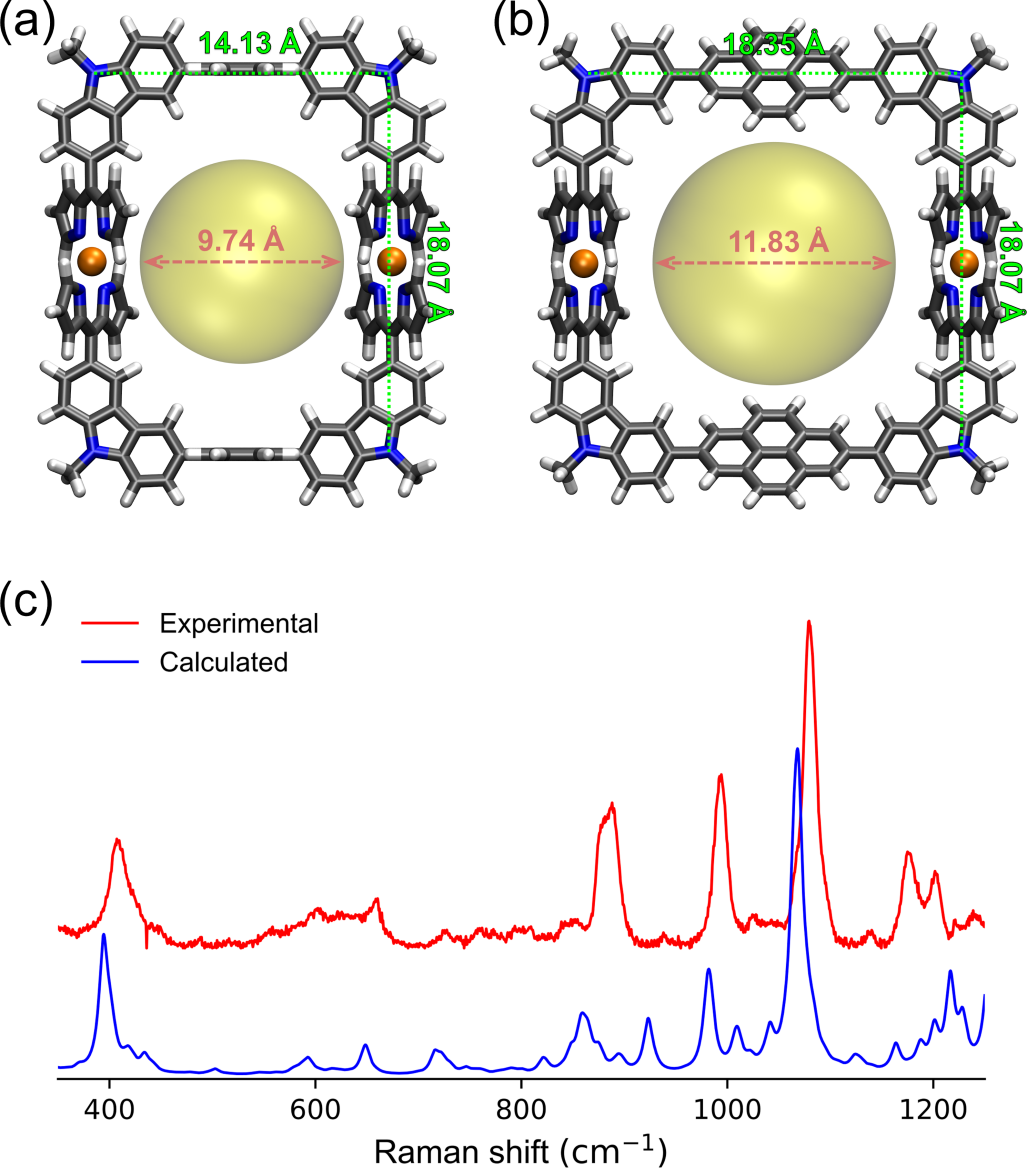
DFT-optimized structure of Cu-nanobrackets (a) **1a** and (b) **1b**. The yellow regions indicate their spherical cavities. (c) Experimental and calculated results of Raman spectra of **1b** (λ_ex_ = 488 nm). For calculation, Raman activity was transferred to Raman intensity (298.15 K, the full width at half maximum (FWHM) is 10 cm^−1^) and the frequency scale factor 0.952 was used for correction [21].

The metal complex of **4b** with copper(II) was prepared, because copper(II) exhibited better extraction and separation abilities than cobalt(II) and palladium(II) in the case of Cu-tethered rectangular nanobrackets **1a** [11]. Before SWNT extraction, the square Cu-nanobrackets **1b** were successfully obtained by the reaction between the nanobracket **4b** and copper(II) acetylacetonate in THF at room temperature. The product was characterized as **1b** by MALDI-TOF mass spectrometry (Figure 1a), absorption (Figure 1b) and Raman (Figure 2c) spectroscopies after chromatographic purification. The mass spectrum clearly indicates that the detected mass number is matched with that of **1b**. In the absorption spectrum, the absorption bands at 300–340 nm and 430 nm can be assigned to pyrene and dipyrrin moieties for **4b**, respectively [18–19]. After complexation, a significant red-shift of the dipyrrin band to 465 nm was observed due to the metal coordination with the dipyrrin moieties in **4b**.

To compare the structures of **1a** and **1b**, they are calculated through geometry optimization with the density functional theory (DFT) method. The alkyl chains at the nitrogen atoms of carbazoles are simplified as methyl groups. As shown in Figure 2a and 2b, while both of them have rectangular structures, the shape of **1b** is almost square. As shown in Figure 2b, the distances between the two nitrogen atoms at carbazoles in **1b** are 18.35 and 18.07 Å along the pyrene and Cu-dipyrrins, respectively. The corresponding distances in **1a** are 14.13 and 18.07 Å along the anthracene and Cu-dipyrrins, respectively, as shown in Figure 2a. Meanwhile, the dihedral angles between pyrene and carbazoles in **1b** are 34.2°, which is much smaller than those of anthracene (89.7°), due to less steric hindrance along the pyrene–carbazole bond than that along the anthracene–carbazole one. To predict the appropriate diameters of SWNTs, the spherical cavity sizes were calculated by considering van der Waals radii of all the atoms. The cavity size of Cu-nanobrackets **1b** (11.83 Å) is 2.09 Å larger than that of **1a** (9.74 Å), implying preference of **1b** to larger diameter of SWNTs.

In the Raman spectra at 488 nm excitation wavelength, **1a** and **1b** show the signals at similar wavenumbers in the range of 400–1200 cm^−1^ (Figure 2c and Figure S1 in Supporting Information File 1). The spectra are almost consistent with the calculated ones. In addition, the four major Raman bands at 405, 880, 990 and 1080 nm are assigned to the corresponding vibration modes shown in Figure S2 (Supporting Information File 1). They are related to the vibrations around the dipyrrin-Cu complexation moieties in **1b**, because the moieties absorbing ≈465 nm light (Figure 1b) should be excited at the resonance Raman spectroscopy using a 488 nm laser. This is supported by the observation that the excitation at 633 and 785 nm where **1b** did not show any absorption (Figure 1b) did not give any Raman signals [20].

### Extraction of SWNTs with Cu-nanobrackets **1b**

We then used the nanobracket **4b** and copper(II) acetylacetonate for the SWNT extraction. Taking the cavity size of Cu-nanobrackets **1b** (Figure 2b) into consideration, we chose HiPco SWNTs (diameter range: 0.7–1.2 nm) as a raw material for the SWNT extraction. Before adding copper(II) acetylacetonate, the nanobracket **4b** and HiPco SWNTs were bath-sonicated for 9 h in 2-propanol. Meanwhile, **4b** is assumed to form complexes with SWNTs. After copper(II) acetylacetonate was added to the mixture, the cyclic Cu-nanobrackets **1b** would be formed to interlock SWNTs with appropriate diameters during further sonication for 15 h. The extract was collected after centrifugation and subjected to the following characterizations.

**Figure 3:**
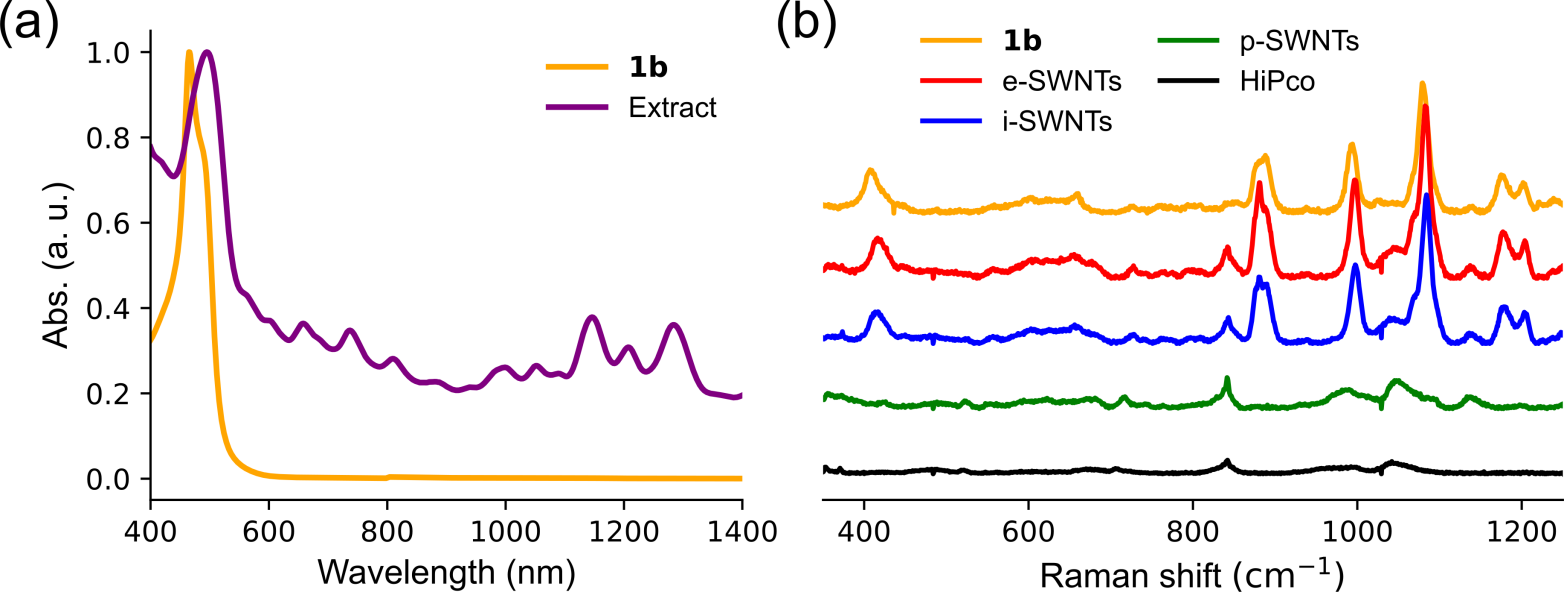
(a) Absorption spectra of Cu-nanobrackets **1b** and SWNT extract; (b) Raman spectra of Cu-nanobrackets **1b**, HiPco SWNTs, extracted, interlocked, and pristine SWNTs, corresponding to e-, i- and p-SWNTs, respectively (λ_ex_ = 488 nm). Raman intensities of all SWNTs are normalized at G-band.

The normalized absorption spectrum of the extract is shown in Figure 3a. Comparing with Cu-nanobrackets **1b**, a significant baseline upward shift is observed, suggesting the existence of SWNTs in the extract. Actually, the absorption bands in the range of 500–1400 nm are attributed to the extracted SWNTs. The absorption band of Cu-nanobrackets **1b** is red-shifted from 465 to 495 nm in the extract, suggesting the complexation of **1b** with SWNTs.

To further investigate whether the interlocked structure is formed, the concentrated extract (e-SWNTs) was washed thoroughly with dichloromethane by sonication and filtration to remove unlocking host molecules. The resulting SWNTs (i-SWNTs) were demetallized with dithiothreitol (DTT) and washed to afford the pristine SWNTs (p-SWNTs). As shown in Figure 3b, all the materials were analyzed with Raman spectroscopy in the solid phase with normalization at G-band (1570 cm^−1^) of SWNTs. The characteristic signals from Cu-nanobrackets **1b** are observed in e- and i-SWNTs, but not in p-SWNTs, indicating the interlocking of SWNTs with **1b**, because the mechanically interlocking cyclic molecules are reported not to be removed with washing [11,22–24]. In addition, these Raman signals of **1b** shift to higher frequencies at e- and i-SWNTs, similarly to our previous report [11], probably due to the structural change of Cu-nanobrackets **1b**, or the energy transfer between the interlocked SWNTs and the cyclic Cu-nanobrackets. After thorough removal of the host molecules with DTT, weak signals are remained at p-SWNTs, known as intermediate frequency modes (IFM) of SWNTs [25]. The IFM signals are also observed in e- and i-SWNTs, showing the coexistence of both **1b** and SWNTs in these samples.

**Figure 4:**
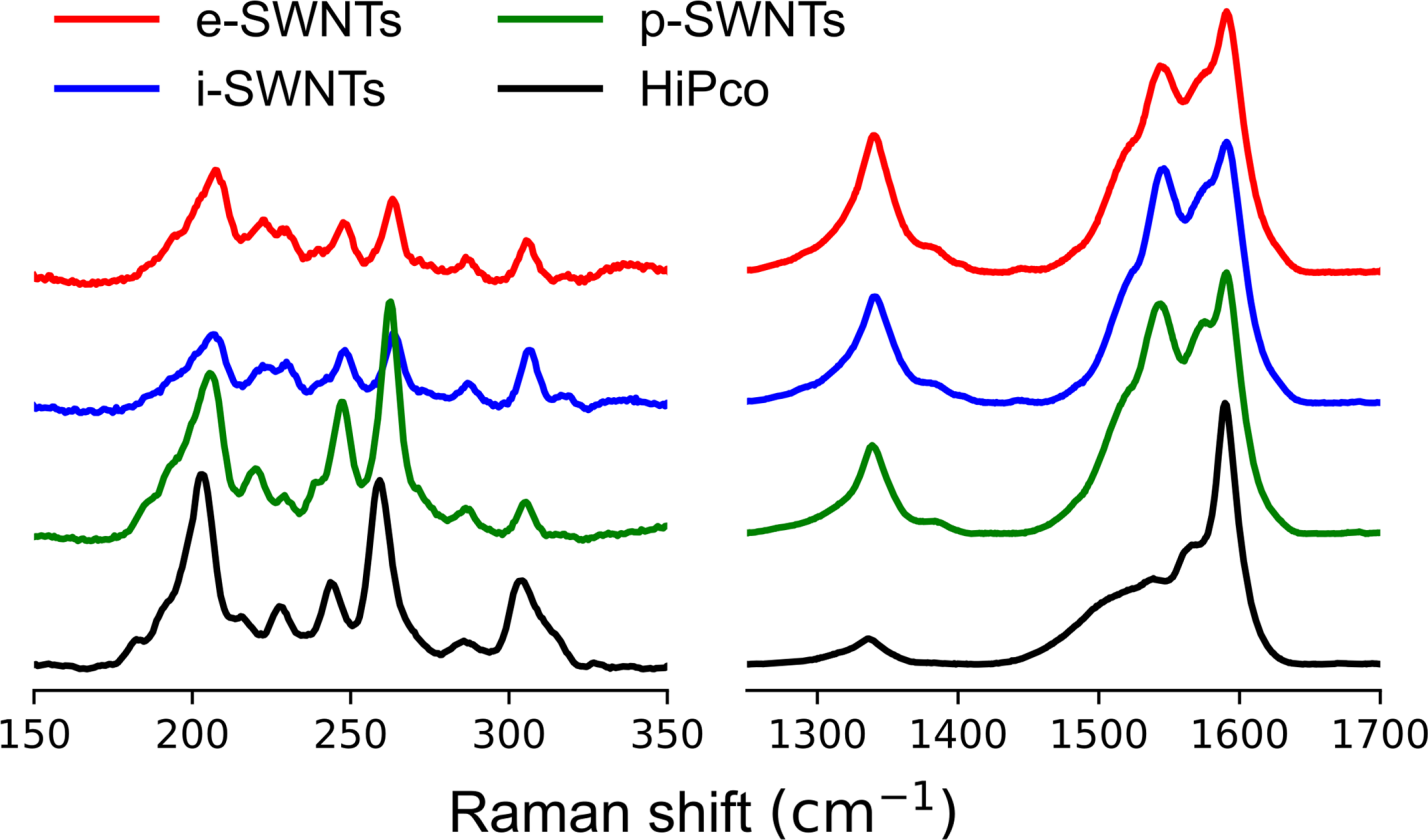
Raman spectra of HiPco SWNTs, e-, i-, and p-SWNTs (λ_ex_ = 488 nm) at RBM and G-band regions. Raman intensities of all SWNTs are normalized at G-band.

The difference in the intensity and Raman shift of the signals in the radial breathing mode (RBM) region would be another evidence for the strong intermolecular interaction between Cu-nanobrackets **1b** and SWNTs. The interlocking of the SWNTs may restrict its radial breathing vibration, resulting in higher frequency shift as shown in Figure 4 [11,26]. With normalization in G-band at 1570 cm^−1^, the relative intensity decreases in the RBM region of e- and i-SWNTs may be due to light absorption and/or peak broadening caused by the interlocking Cu-nanobrackets **1b**.

### Characterization of the extracted SWNTs

**Figure 5:**
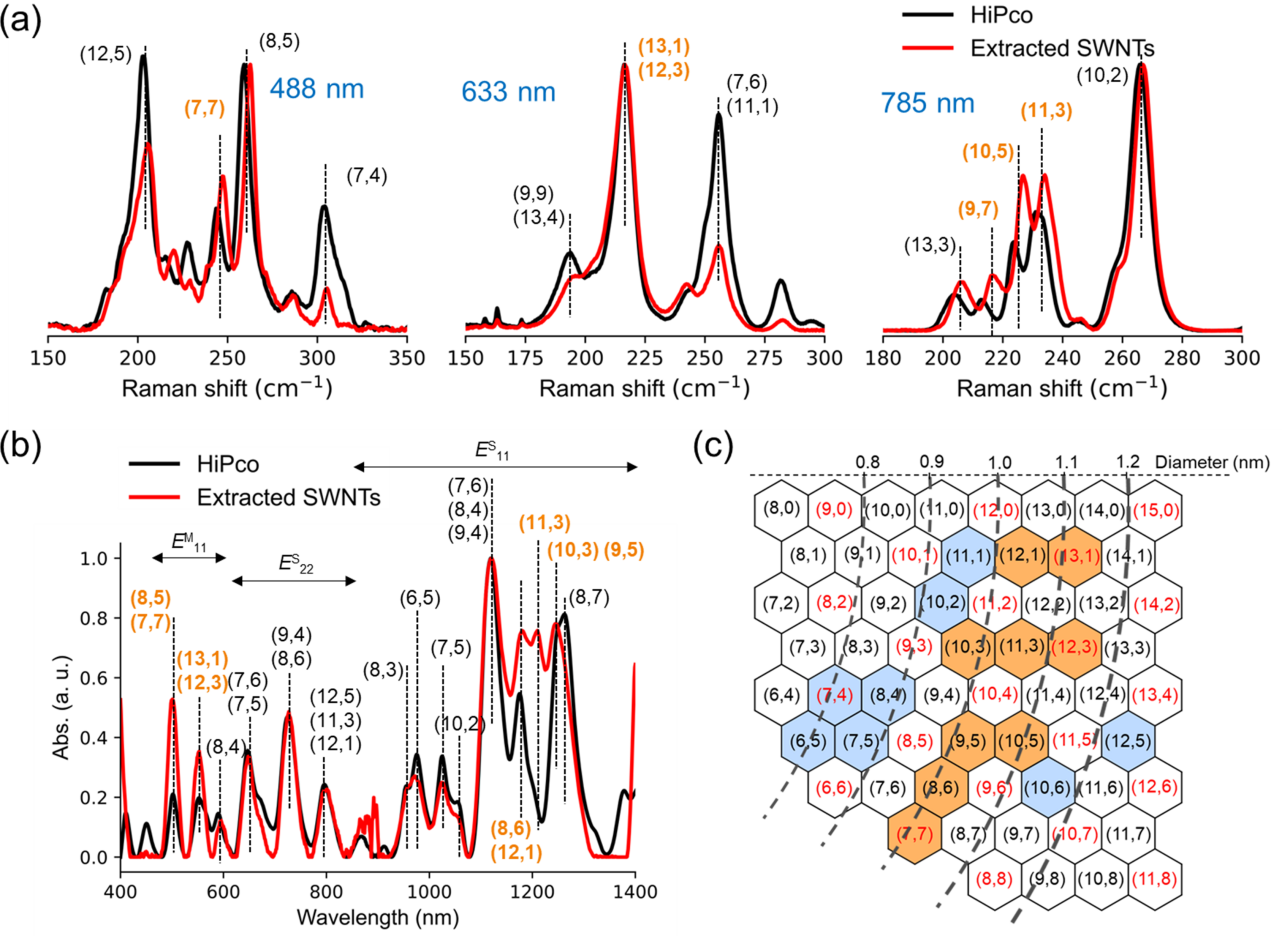
(a) Raman spectra of HiPco and extracted SWNTs at 488, 633 and 785 nm excitation wavelengths, normalized at 260, 216 and 266 cm^−1^, respectively, after baseline correction; (b) normalized absorption spectra of HiPco and extracted SWNTs after dispersing with SDBS in D_2_O after baseline correction; (c) summary of increase (orange) and decrease (blue) in (*n*,*m*)-SWNT abundance in the p-SWNTs. Red and black characters indicate metallic and semiconducting SWNTs, respectively. (a, b) Enriched (*n*,*m*)-SWNTs are shown in orange.

To evaluate the diameter enrichment of the extracted SWNTs, Raman spectra are measured at 488, 633 and 785 nm excitation wavelengths for the p-SWNTs. In the RBM region, the Raman shift of the signal is known to be inversely proportional to the diameter of SWNTs. Even though the intensity of the Raman peak is not fully proportional to the content of the corresponding SWNTs, the relative abundance can be estimated through spectral change at the RBM. In Figure 5a, (*n*,*m*)-SWNTs are assigned according to the references [27–28]. At 488, 633 and 785 nm excitation wavelengths, (7,7)-, (13,1)-, (12,3)-, (9,7)-, (10,5)-, and (11,3)-SWNTs shown in orange in Figure 5a, corresponding to 0.96–1.10 nm in diameters, were relatively enriched after the extraction.

The p-SWNTs were dispersed in 1% SDBS solution in D_2_O through probe-type sonication followed by ultracentrifugation at 136000*g*. The absorption spectrum of the supernatant was measured to evaluate the diameter enrichment. As shown in Figure 5b, (8,6)-, (12,1)-, (11,3)-, (10,3)-, and (9,5)-SWNTs in the semiconducting region (*E*^S^_11_) and (8,5)-, (7,7)-, (13,1)-, (12,3)-SWNTs in the metallic region (*E*^M^_11_) are enriched as summarized in a graphene map (Figure 5c). This shows that Cu-nanobrackets **1b** preferentially recognize larger diameters with wider range (0.94–1.10 nm) than **1a** (0.90–0.92 nm) due to the larger cavity with more flexibility of **1b** than **1a**.

### Theoretical calculations

To interpret the diameter selectivity, the binding energy between SWNTs and the host molecules is calculated by the GFN2-xTB method, because the number of atoms is too large to use DFT methods. The binding energy *E*_bind_ is calculated by the following equation:


[1] $$E_{\text{bind}}=E_{\text{complex}}-E_{\text{host}}-E_{\text{SWNTs}}$$

**Figure 6:**
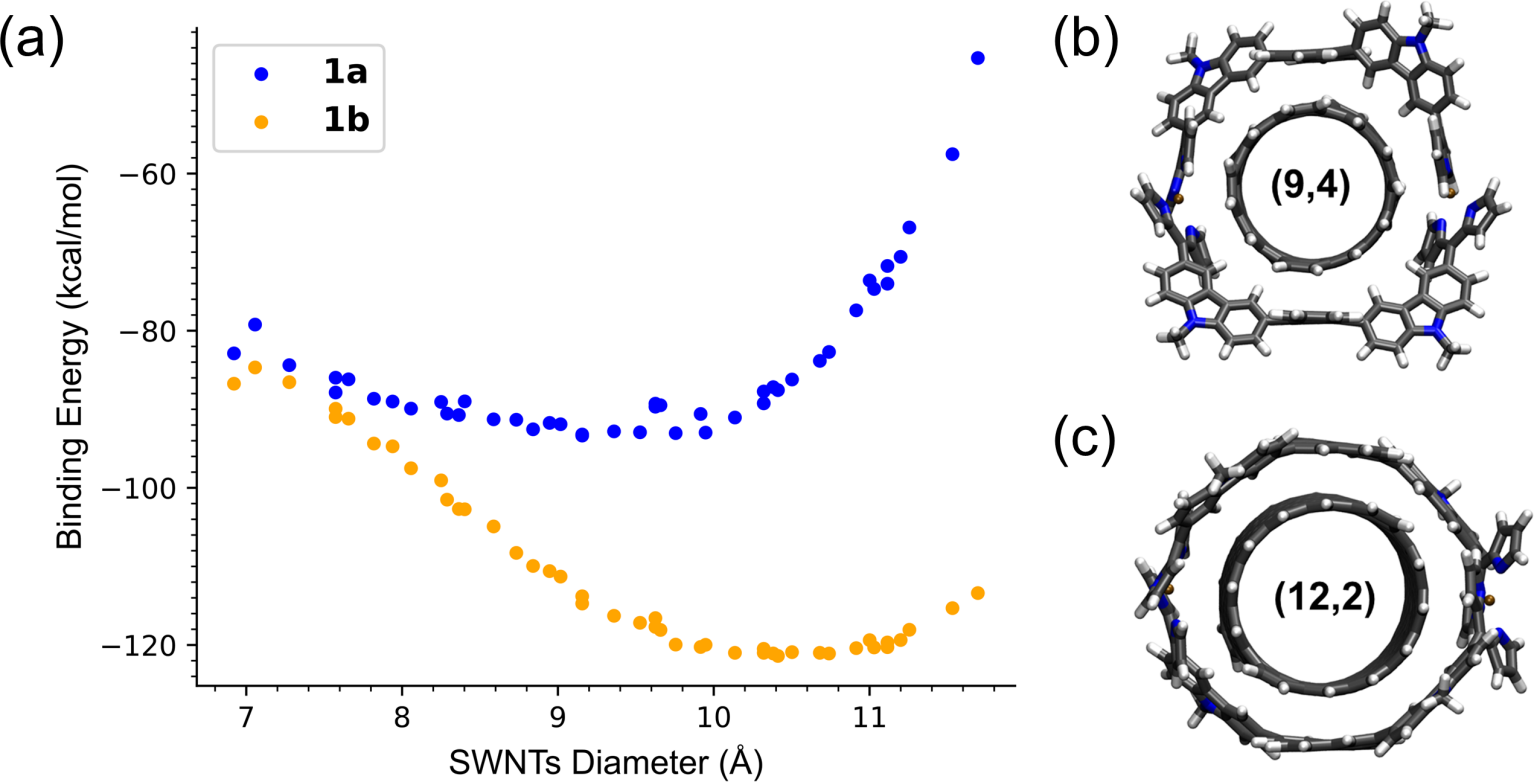
(a) Binding energy between SWNTs of various (*n*,*m*)-structures with Cu-nanobrackets **1a** and **1b**; GFN2-xTB-optimized structures of (b) **1a**@(9,4)-SWNT and (c) **1b**@(12,2)-SWNT complexes.

The *E*_host_, *E*_SWNTs_ and *E*_complex_ are the electronic energies of the host molecules, SWNTs and their complexes with interlocking structures, respectively, after the geometry optimization with the GFN2-xTB method. The 45 different kinds of SWNTs with length of ≥3.0 nm and diameter range of 0.7–1.2 nm are chosen for the calculation (Table S1, Supporting Information File 1). The relationship between the SWNT diameter and *E*_bind_ is shown in Figure 6a. Even though the *E*_bind_ magnitude of van der Waals-dominated complexes of conjugated π-systems (e.g., fullerenes) are sometimes overestimated in the calculation by GFN2-xTB [16], the *E*_bind_ can be compared in the SWNT complexes with Cu-nanobrackets **1a** and **1b**. (9,4)- and (12,2)-SWNTs with diameters of 0.92 and 1.04 nm gave the largest *E*_bind_ values with **1a** and **1b**, respectively. According to our previous report, Cu-nanobrackets **1a** selectively extracted (8,5)-, (7,6)-, and (9,4)-SWNTs with a diameter range of 0.90–0.92 nm from the HiPco SWNTs [11], which is consistent with the above calculation result.

In the optimized structures of **1a**@(9,4)-SWNT and **1b**@(12,2)-SWNT complexes (Figure 6b and 6c, respectively), while CH–π interactions of the four carbazoles with the SWNT surface stabilize the former complex, the carbazoles facing to the SWNT surface suggest π–π interactions in the latter complex. Their difference in *E*_bind_ shown in Figure 6a can be interpreted by that in these interactions. In addition, their difference in conformational flexibility supports the wider favorable diameter range of **1b** than **1a**.

## Conclusion

A cyclic host molecule (Cu-nanobrackets **1b**) tethered by copper(II) dipyrrin linkage was designed, synthesized, and applied to SWNT separation. Cu-nanobrackets **1b** were in situ formed by interlocking SWNTs to disperse them in 2-propanol. Raman and absorption spectroscopies reveal the diameter enrichment of several kinds of SWNTs in the diameters ranging from 0.94 to 1.10 nm among ≈20 kinds of SWNTs from 0.76 to 1.20 nm in their diameters. The most preferable SWNTs for **1b** are found to be 1.04 nm in diameter by theoretical calculations, supporting the experimental results.

## Experimental

### Materials and instruments

Reagents were purchased from Sigma-Aldrich Co., Nacalai Tesque Co., Tokyo Chemical Industry Co., Ltd., FUJIFILM Wako Pure Chemical Industries, Ltd. All organic solvents are purchased from Nacalai Tesque Co., FUJIFILM Wako Pure Chemical Industries, Ltd. and were used without purification unless indicated otherwise. The silica gel 60 N (spherical, neutral) was purchased from Kanto Chemical Co., Ltd. All reactions were performed in oven-dried glassware unless stated otherwise and were monitored by TLC using 0.25 mm silica gel plates with UV indicator (70F-254). Commercial HiPco (purchased from Carbon Nanotechnologies Inc., with diameter 0.8– 1.2 nm) were used.

For centrifugal operation, Avanti J-E of Beckman Coulter and Hitachi Himac CS 150 GC II were used. Mass spectra were recorded on Bruker Autoflex III using the matrix-assisted laser desorption/ionization time-of-flight (MALDI-TOF) method with anthracene as the matrix for all measurements and analyzed with mMass [29] software. ^1^H and ^13^C NMR spectra were recorded on JNM-ECS 400 spectrometer using the sample solutions in chloroform-*d* (CDCl_3_). Raman spectra were recorded on LabRam HR800 (Horiba Ltd.) and take the average of more than ten different spots for the final curve. UV–Vis–NIR spectra were recorded on a Shimadzu UV3600 spectrophotometer. PR-1 (THINKY) and Astrason XL 2020, 550 W was used for bath-sonication and prob type sonication, respectively.

### Extraction of SWNTs

In a manner similar to [11], the pyrene-based nanobracket **4b** (10 mg) and SWNTs (HiPco, 10 mg) in 2-propanol (40.0 mL) were bath-sonicated with rotation by PR-1 (THINKY) for 9 h with six cycles of 85 and 5 min with and without sonication, respectively. After copper(II) acetylacetonate (4.0 mg) was added, the mixture was bath-sonicated with rotation for 15 h with 10 of the same cycles. SWNTs extracts were obtained by centrifugation at 50400*g* for 16 h and subjected to UV−vis−NIR measurements (Figure 3a). After concentrating the extract obtained above, the residue (e-SWNTs) was suspended in dichloromethane by sonication and filtered through a PTFE membrane (pore size 0.1 μm). This washing process was repeated three times, until no absorption bands from the host molecules were observed in the UV−vis spectra of washings. The resulting i-SWNTs were analyzed with Raman spectroscopy at 488 nm. The i-SWNTs were bath-sonicated with dithiothreitol (50 mg) in dichloromethane for 30 min followed by filtration with a PTFE membrane. This process to remove host molecules was repeated three times, until no absorption bands from the host molecules were observed in the UV−vis spectra of washings. After the resulting p-SWNTs were washed with dichloromethane several times to thoroughly remove all the organic molecules, the solid sample was analyzed with Raman spectroscopy at excitations of 488, 633, and 785 nm (Figure 5a). After the solid sample was dispersed in D_2_O (12.0 mL) in the presence of SDBS (10 mg mL^−1^) by bath sonication with rotation at 20 °C for 18 h and centrifuged at 136000*g* for 40 min, the upper layer (about 85%) of the supernatant was poured out and subjected to UV−vis−NIR measurements (Figure 5b). For the dispersion of raw HiPco SWNTs, probe-type sonication was carried out below 0 °C for 2 h, followed by centrifugation at 543000*g* for 50 min. The supernatant was subjected to UV−vis−NIR measurements (Figure 5b).

### Computational methods

The geometry optimization and frequency analysis of Cu-nanobrackets **1a** and **1b** were performed at (U)ωB97X-D/6-31+G(d,p)-SDD(Cu) [30–33] level using the Gaussian 16 program [34], with calculating the pre-resonance Raman activities at 488 nm excitation wavelength. The plotting of Raman spectra and calculation of the molecular cavity were accomplished with the help of Multiwfn [35]. VMD [36] was used for the modeling of (*n*,*m*)-SWNTs and the structure visualization. Molclus [37] was used for the modeling of Cu-nanobrackets@SWNTs complexes. The binding energies of Cu-nanobrackets@SWNTs complexes were computed with the GFN2-xTB method by using the xtb software [16–17].

## Supporting Information

File 1 Details of theoretical calculations, experimental procedures, copies of ^1^H and ^13^C NMR spectra.

## Data Availability

All data that supports the findings of this study is available in the published article and/or the supporting information to this article.
