# Supplementary material for: Diameter-selective extraction of single-walled carbon nanotubes by interlocking with Cu-tethered square nanobrackets
Source: Beilstein J Org Chem. 2024 Jun 5;20:1298–307. doi: 10.3762/bjoc.20.113 (PMC11181202; doi:10.3762/bjoc.20.113)
Supplement: File 1 — Details of theoretical calculations, experimental procedures, copies of 1H and 13C NMR spectra. [file Beilstein_J_Org_Chem-20-1298-s001.pdf]

BEILSTEIN JOURNAL OF ORGANIC CHEMISTRY

Supporting Information

for

Diameter-selective extraction of single-walled carbon nanotubes by interlocking with Cu-tethered square nanobrackets

Guoqing Cheng and Naoki Komatsu

Beilstein J. Org. Chem. **2024,** *20,* 1298–1307. doi:10.3762/bjoc.20.113

Details of theoretical calculations, experimental procedures, copies of ^{1}H and ^{13}C NMR spectra

License and Terms: This is a supporting information file under the terms of the Creative Commons Attribution License (https://creativecommons.org/licenses/by/4.0). Please note that the reuse, redistribution and reproduction in particular requires that the author(s) and source are credited and that individual graphics may be subject to special legal provisions.
The license is subject to the *Beilstein Journal of Organic Chemistry* terms and conditions: (https://www.beilstein-journals.org/bjoc/terms)

Calculation of the pre-resonance Raman spectra

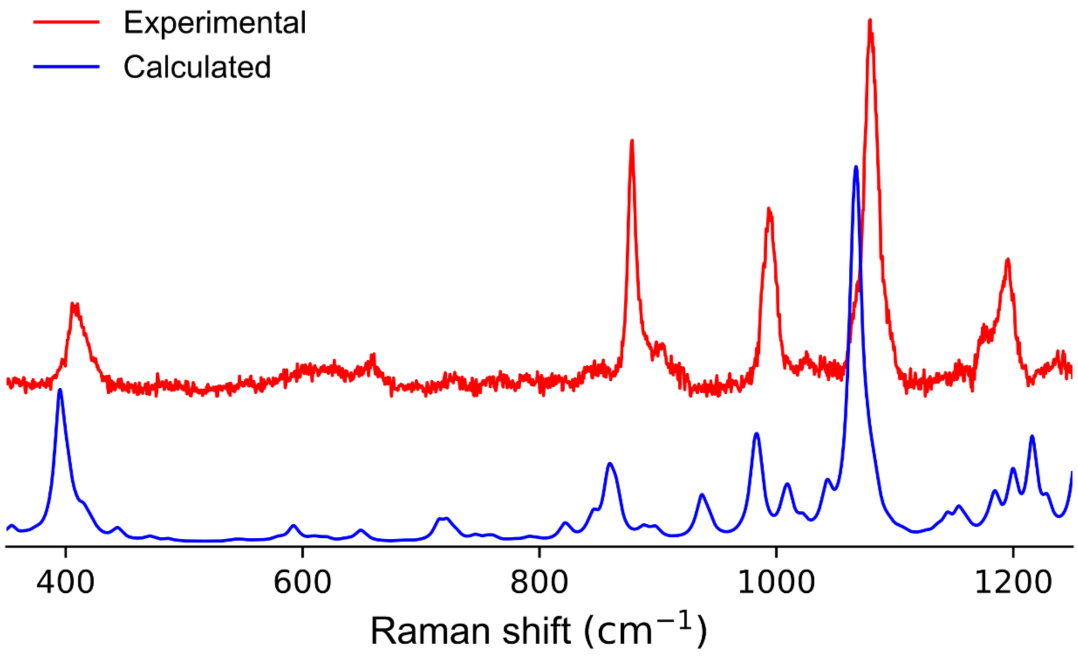

Figure S1: Experimental and calculated Raman spectra of Cu-nanobrackets **1a** (λ_{ex} = 488 nm, 298.15 K, FWHM = 10 cm^{-1}).

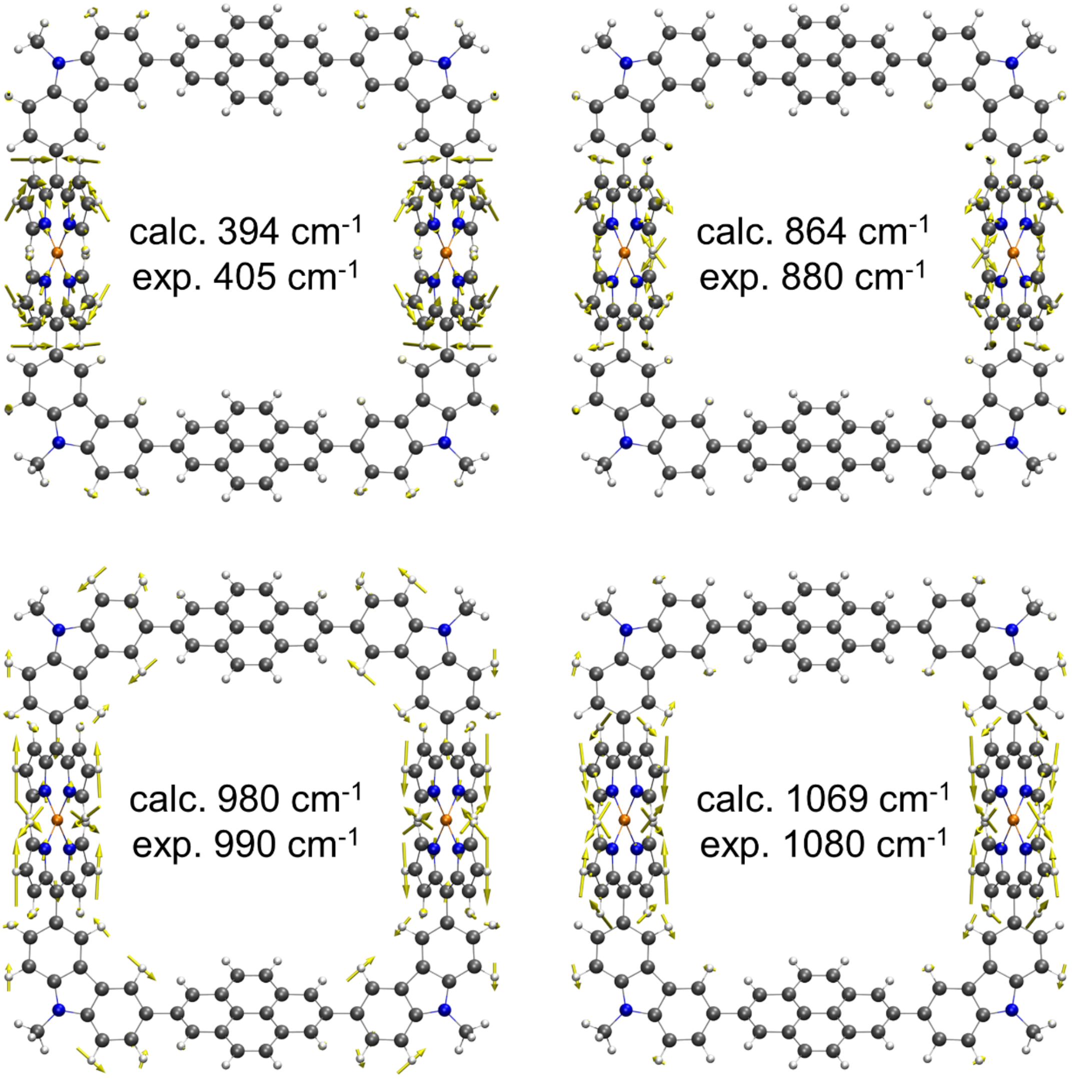

Figure S2: Selected vibration modes of Cu-nanobrackets **1b** with relatively large Raman activities are indicated by the displacement vectors as yellow-colored arrows. The calculated frequencies are corrected with the scale factor (0.952).

Calculation of the binding energies between Cu-nanobrackets and SWNTs

For the calculation of the binding energy between Cu-nanobrackets and SWNTs, totally 45 kinds of SWNTs covering the main components in HiPco SWNTs with more than 3 nm length are generated by VMD and the terminals of these SWNTs are capped by hydrogen atoms. Geometry optimizations of three different conformations of each Cu-nanobrackets@(*n*,*m*)-SWNT complex generated by Molclus software are carried out with GFN2-xTB method by using xtb software with the default convergence level. Among these conformations, the one with the lowest energy is used for further analysis. The results were summarized in Table S1.

Table S1: E_{bind} of Cu-nanobrackets **1a** and **1b** with (*n*,*m*)-SWNTs.

n	*m*	E_{bind} (kcal/mol)		SWNT	*n*	*m*	E_{bind} (kcal/mol)		SWNT
		**1a**	**1b**	Diameter (Å)			**1a**	**1b**	Diameter (Å)
10	0	-89.01	-94.72	7.94	6	4	-82.89	-86.75	6.92
11	0	-91.33	-108.28	8.73	7	4	-86.19	-91.19	7.66
12	0	-92.93	-117.18	9.53	8	4	-88.99	-102.73	8.40
13	0	-89.25	-121.00	10.32	9	4	-93.35	-114.73	9.16
14	0	-74.02	-119.68	11.11	10	4	-90.60	-120.25	9.92
9	1	-87.87	-90.99	7.57	11	4	-83.84	-121.00	10.68
10	1	-90.73	-102.69	8.36	6	5	-85.98	-89.93	7.57
11	1	-93.21	-113.80	9.16	7	5	-90.55	-101.51	8.29
12	1	-92.97	-119.98	9.95	8	5	-91.90	-111.29	9.02
13	1	-82.71	-121.09	10.74	9	5	-93.04	-119.95	9.76
14	1	-57.53	-115.31	11.53	10	5	-86.21	-120.91	10.50
8	2	-84.38	-86.55	7.28	11	5	-66.86	-118.06	11.26
9	2	-89.92	-97.51	8.06	6	6	-89.07	-99.05	8.25
10	2	-92.55	-109.94	8.84	7	6	-91.73	-110.60	8.95
11	2	-89.68	-117.70	9.63	8	6	-89.49	-118.09	9.66
12	2	-87.56	-121.39	10.41	9	6	-87.18	-121.08	10.38
13	2	-70.60	-119.37	11.20	10	6	-71.76	-120.28	11.11
7	3	-79.22	-84.68	7.06	7	7	-89.28	-116.58	9.63
8	3	-88.66	-94.36	7.82	8	7	-87.73	-120.49	10.32
9	3	-91.28	-104.91	8.59	9	7	-74.69	-120.31	11.03
10	3	-92.81	-116.29	9.36	8	8	-73.61	-119.38	11.00
11	3	-91.05	-121.00	10.14	9	8	-45.30	-113.39	11.70
12	3	-77.41	-120.40	10.91					

Synthesis and characterization

Synthesis of 6,6'-(pyrene-2,7-diyl)bis(9-octyl-9H-carbazole-3-carbaldehyde) (3b)

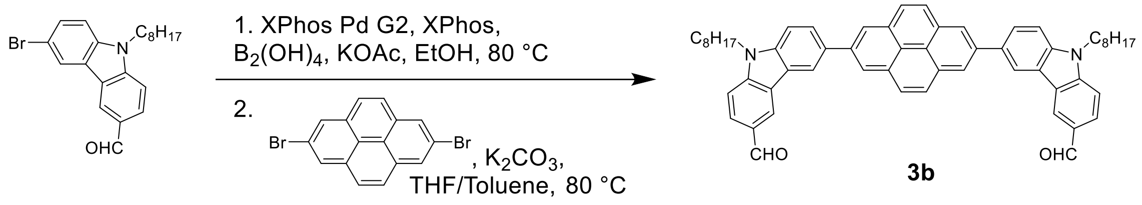

XPhos Pd G2 (19.7 mg, 0.025 mmol), XPhos (23.5 mg, 0.050 mmol), tetrahydroxydiboron (449 mg, 5.0 mmol), potassium acetate (494 mg, 5.0 mmol) and 3-bromo-*N*-octyl-6-formylcarbazole [1] (538 mg, 1.4 mmol) were dissolved in EtOH (15 mL). The flask was then purged by Ar, after O_2 was removed under the vacuum. After stirring at 80 °C for 3 h, aqueous potassium carbonate (1.8 M, 2.5 mL) with the following 2,7-dibromopyrene (180 mg, 0.50 mmol) dispersed in a dry mixture of THF (12 mL) and toluene (4.0 mL) were added into the reaction mixture. The reaction was deoxygenated again, stirred at 80 °C overnight. After cooling to room temperature, H_2O (20 mL) was added. The aqueous layer was extracted by DCM three times and the combined organic layers were dried over $MgSO_4$, concentrated under vacuum, and washed with hexane/DCM (1:1) followed by recrystallization in hexane/DCM to give **3b** as white solid (303 mg, yield: 75 %). MALDI TOF-MS (matrix: anthracene) of [**3b**]$^+$: $C_{58}H_{56}N_2O_2$ Calc. m/z: 812.4342, found m/z: 812.4505. ^{1}H NMR (400 MHz, CHLOROFORM-*D*) δ 10.15 (s, 2H), 8.77 (d, *J* = 1.6 Hz, 2H), 8.69 (d, *J* = 1.6 Hz, 2H), 8.54 (s, 4H), 8.22 (s, 4H), 8.12 – 8.04 (m, 4H), 7.65 (d, *J* = 8.4 Hz, 2H), 7.54 (d, *J* = 8.5 Hz, 2H), 4.42 (t, *J* = 7.3 Hz, 4H), 2.08 – 1.88 (m, 4H), 1.50 – 1.19 (m, 20H), 0.88 (t, *J* = 6.9 Hz, 6H). ^{13}C NMR (126 MHz, CHLOROFORM-*D*) δ 191.8, 144.6, 140.8, 139.3, 134.1, 131.6, 128.7, 128.0, 127.0, 124.0, 123.8, 123.6, 123.3, 120.1, 109.9, 109.2, 43.7, 31.8, 29.4, 29.2, 29.0, 27.3, 22.6, 14.1.

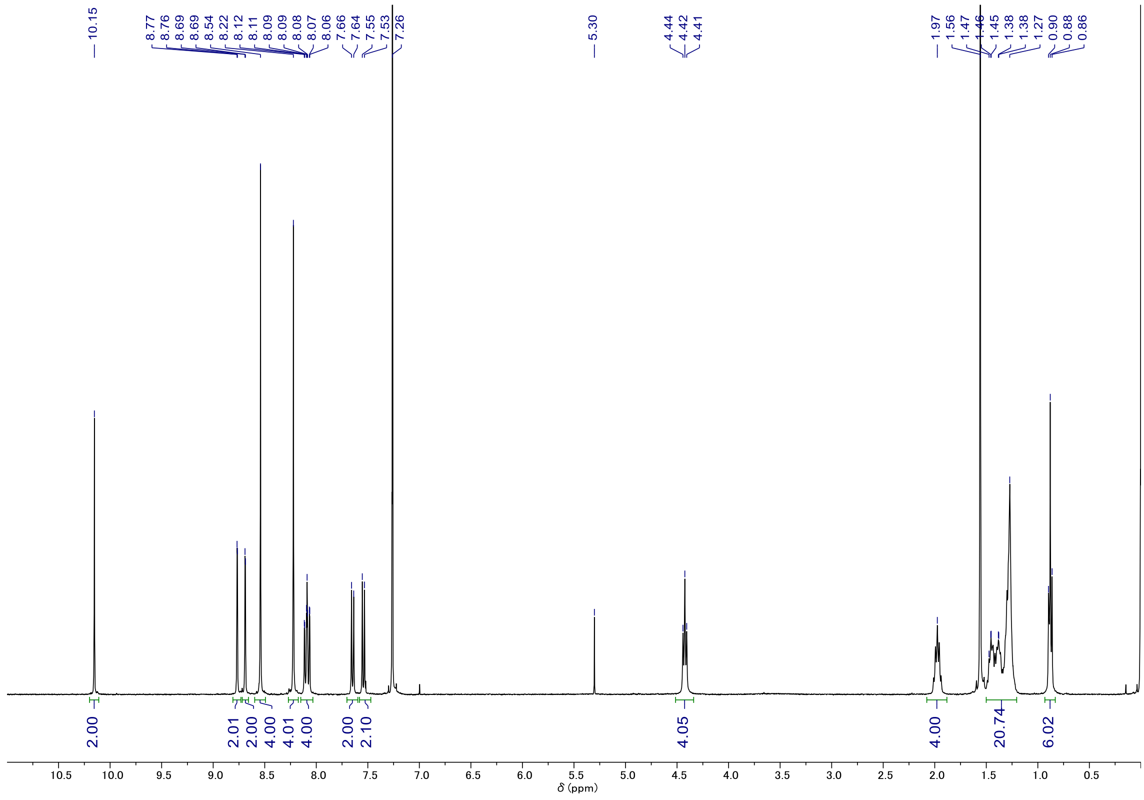
10.15
8.77
8.76
8.69
8.69
8.54
8.22
8.12
8.11
8.09
8.09
8.08
8.07
8.06
7.66
7.64
7.55
7.53
7.26
5.30
4.44
4.42
4.41
1.97
1.56
1.47
1.46
1.45
1.38
1.38
1.27
0.90
0.88
0.86
2.00
2.01
2.00
4.00
4.01
4.00
2.00
2.10
4.05
4.00
20.74
6.02
10.5 10.0 9.5 9.0 8.5 8.0 7.5 7.0 6.5 6.0 5.5 5.0 4.5 4.0 3.5 3.0 2.5 2.0 1.5 1.0 0.5
δ (ppm)

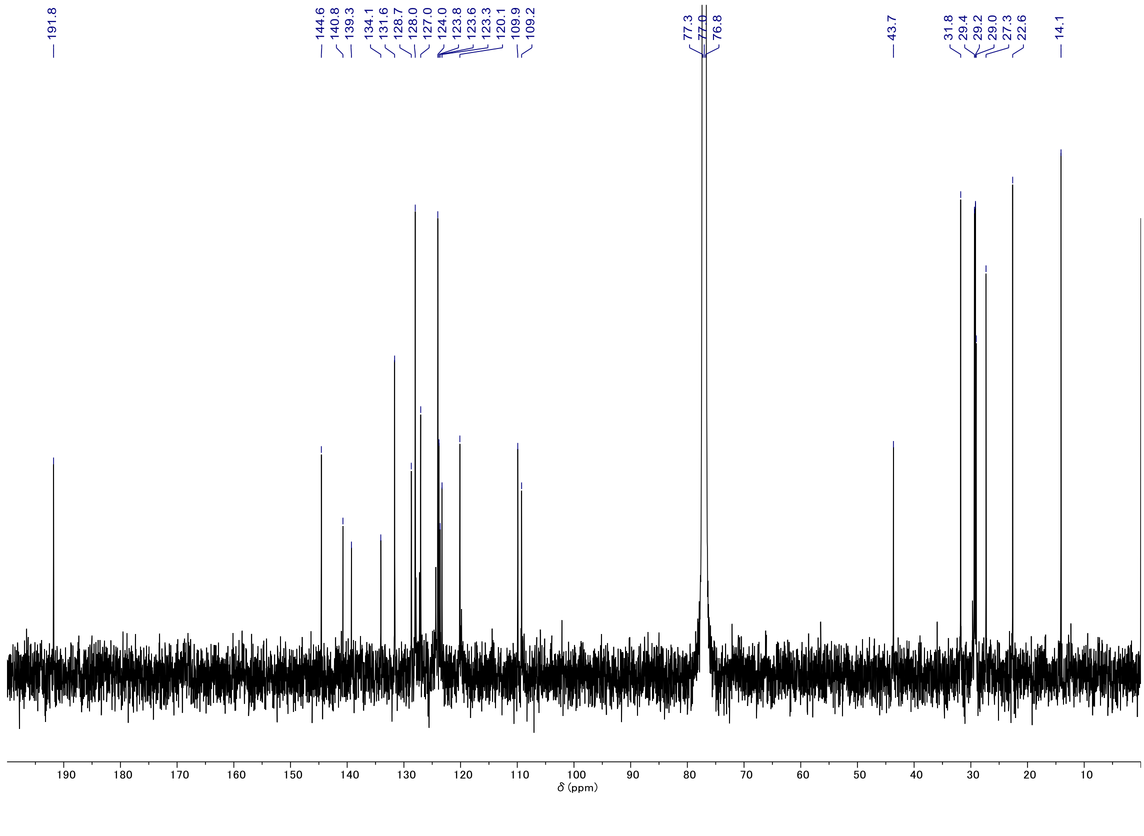
191.8
144.6
140.8
139.3
134.1
131.6
128.7
128.0
127.0
124.0
123.8
123.6
123.3
120.1
109.9
109.2
77.3
77.0
76.8
43.7
31.8
29.4
29.2
29.0
27.3
22.6
14.1
190 180 170 160 150 140 130 120 110 100 90 80 70 60 50 40 30 20 10
δ (ppm)

Synthesis of 4b

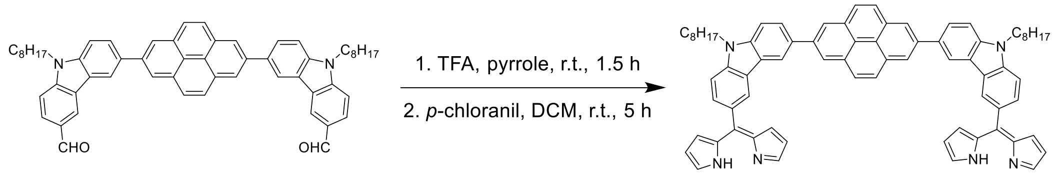

Diadehyde **3b** (220 mg, 0.27 mmol) was dissolved in dry pyrrole (20 mL). After trifluoroacetic acid (20 μL) was added, the mixture was stirred at room temperature for 1.5 h under Ar. The reaction mixture was then diluted with DCM (20 mL), washed with aqueous NaOH (0.10 M, 20 mL) solution and brine, dried over $MgSO_4$, concentrated under vacuum, and washed with hexane/DCM (1/1). Without further purification, the resulting solid (60 mg, 0.058 mmol) was dissolved in DCM (10 mL) and stirred in an ice bath, after which *p*-chloranil (43 mg, 0.17 mmol) in DCM (10 mL) was added dropwise. By stirring at room temperature for 5 h, the reaction mixture was poured over a silica-gel column and eluted with 3% MeOH/DCM. After recrystallization from DCM/hexane, **4b** was obtained as an orange solid (20 mg, total yield: 7.3%). MALDI TOF-MS (matrix: anthracene) of [**4b**]$^+$: $C_{74}H_{68}N_6$ Calc. m/z: 1040.5505, found m/z: 1040.5958. ^{1}H NMR (400 MHz, CHLOROFORM-*D*) δ 8.62 (s, 2H), 8.51 (s, 4H), 8.40 (s, 2H), 8.17 (s, 4H), 8.12 – 8.07 (m, 2H), 7.90 (s, 4H), 7.72 – 7.62 (m, 4H), 7.54 (d, *J* = 8.5 Hz, 2H), 6.95 – 6.80 (m, 4H), 6.68 – 6.50 (m, 4H), 4.45 (t, *J* = 7.1 Hz, 4H), 2.06 – 1.97 (m, 4H), 1.54 – 1.23 (m, 22H), 0.89 (t, *J* = 6.7 Hz, 6H). ^{13}C NMR (126 MHz, CHLOROFORM-*D*) δ 142.7, 142.6, 142.5, 140.8, 139.3, 133.8, 133.2, 131.6, 128.0, 128.0, 127.0, 124.0, 123.6, 123.4, 120.2, 117.2, 109.9, 43.7, 31.8, 29.4, 29.2, 29.2, 27.4, 22.7, 14.1.

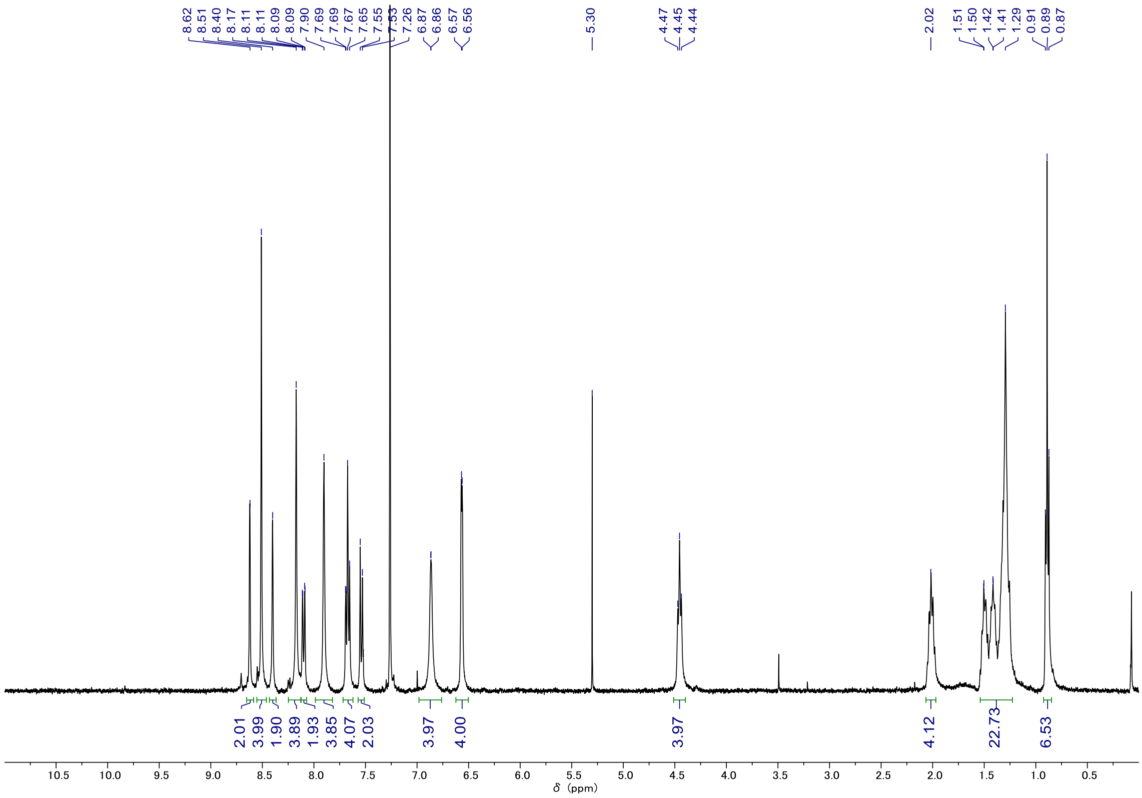

8.62
8.51
8.40
8.17
8.11
8.11
8.09
8.09
7.90
7.69
7.69
7.67
7.65
7.55
7.53
7.26
6.87
6.86
6.57
6.56
5.30
4.47
4.45
4.44
2.02
1.51
1.50
1.42
1.41
1.29
0.91
0.89
0.87
2.01
3.99
1.90
3.89
1.93
3.85
4.07
2.03
3.97
4.00
3.97
4.12
22.73
6.53
δ (ppm)

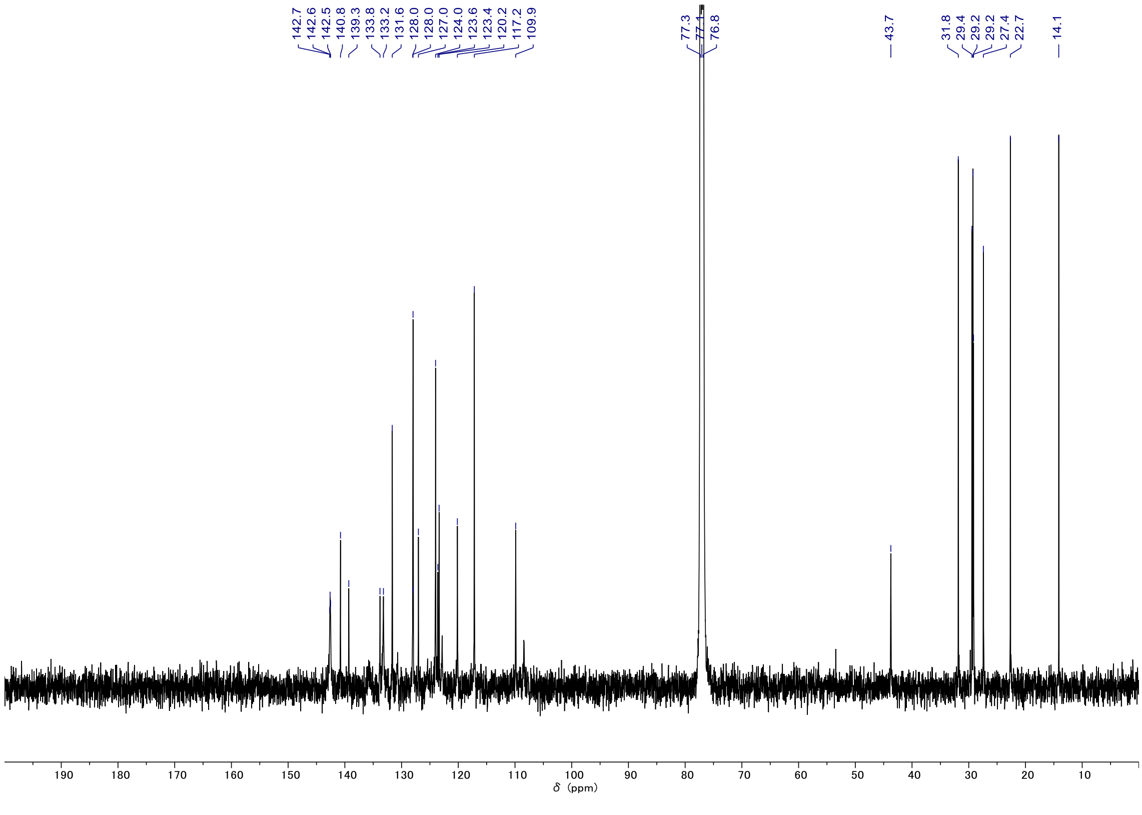

142.7
142.6
142.5
140.8
139.3
133.8
133.2
131.6
128.0
128.0
127.0
124.0
123.6
123.4
120.2
117.2
109.9
77.3
77.1
76.8
43.7
31.8
29.4
29.2
29.2
27.4
22.7
14.1
δ (ppm)

Synthesis of Cu-nanobrackets 1b

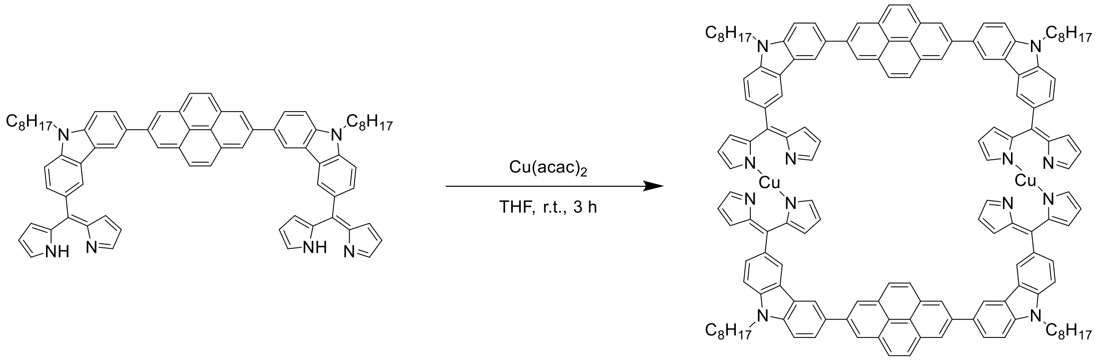

Bis(dipyrrin) **4b** (2.0 mg) and Cu(acac)$_2$ (copper(II) acetylacetonate, 0.75 mg) were dissolved in THF (5.0 mL) and stirred at rt for 3 h. The reaction mixture was concentrated under vacuum and purified by column chromatography (DCM) to afford Cu-nanobrackets **1b** (0.60 mg, 28%). MALDI TOF-MS (matrix: anthracene) of [**1b**]$^+$: $C_{148}H_{132}Cu_2N_{12}$ Calc. *m*/*z*: 2204.9316, found *m*/*z*: 2204.9322.

Reference

1. Chen, H.; Cai, X. R.; Xu, Z. G.; Zhang, T.; Song, B. F.; Li, Y.; Jiang, Q.; Xie, M. G. *Polymer Bulletin* **2008**, *60* (4), 581-590. doi:10.1007/s00289-008-0890-1
